# Reviewer Acknowledgment 2014

**DOI:** 10.1186/s40101-015-0044-8

**Published:** 2015-02-24

**Authors:** Akira Yasukouchi

**Affiliations:** Department of Physiological Anthropology, Faculty of Design, Kyushu University, 4-9-1, Shiobaru, Minami-ku, Fukuoka 815-8540 Japan

## Abstract

The editors of *Journal of Physiological Anthropology* would like to thank all our reviewers who have contributed to the journal in Volume 33 (2014).

**Daijiro Abe**

Japan

**Yasuyo Abe**

Japan

**Vicent Balanza**

Spain

**Kim Boseong**

Korea, South

**Stephen Cheung**

Canada

**Seong**-**Woo Choi**

Korea, South

**Yoon Gi Chung**

Korea, South

**Douglas E. Crews**

United States of America

**Jon Doan**

Canada

**Jeanne Duffy**

United States of America

**Miranda Farage**

United States of America

**Katsunori Fujii**

Japan

**Yuki Fujita**

Japan

**Katsuo Fujiwara**

Japan

**Yoshiyuki Fukuoka**

Japan

**Anuradha Godavarty**

United States of America

**Elena Godina**

Russian Federation

**Kazushige Goto**

Japan

**Demetrios Halazonetis**

Greece

**Hajime Harada**

Japan

**Tetsuo Harada**

Japan

**Nobuko Hashiguchi**

Japan

**Kazutoshi Hayase**

Japan

**Naoyuki Hayashi**

Japan

**Shigekazu Higuchi**

Japan

**Masahiro Horiuchi**

Japan

**Norio Hotta**

Japan

**Qianchuan Huang**

China

**Tomoko Ichinose**

Japan

**Miho Igarashi**

Japan

**Kaoru Inoue**

Japan

**Keita Ishibashi**

Japan

**Koichi Iwanaga**

Japan

**Xanne Janse de Jonge**

Australia

**Emmanuel John**

United States of America

**Colleen Julian**

United States of America

**Myung**-**Chul Jung**

Korea, South

**Kenichi Kaneko**

Japan

**Tetsuo Katsuura**

Japan

**Jooyoung Kim**

United States of America

**Sung**-**Phil Kim**

Korea, South

**Kenta Kimura**

Japan

**Herm Klaus**-**Peter**

Germany

**Hiromitsu Kobayashi**

Japan

**Shunsaku Koga**

Japan

**Kentaro Kotani**

Japan

**Katsuyasu Kouda**

Japan

**Tomoaki Kozaki**

Japan

**Yosuke Kusano**

Japan

**Izelle Labuschagne**

Australia

**Bae Hwan Lee**

Korea, South

**Jeong Beom Lee**

Korea, South

**Joo Young Lee**

Korea, South

**Soomin Lee**

Japan

**Wenbo Luo**

China

**Liang Ma**

China

**Sara Mandelli**

Italy

**Joseph Maté**

Australia

**Maija Miettinen**

Finland

**Byung**-**Chan Min**

Korea, South

**Yoshifumi Miyazaki**

Japan

**Nobuaki Mizuguchi**

Japan

**Takuya Morishita**

Japan

**Takeshi Morita**

Japan

**Midori Motoi**

Japan

**Yuki Motomura**

Japan

**Hideyuki Mukae**

Japan

**Satoshi Muraki**

Japan

**Harunobu Nakamura**

Japan

**Masashi Nakamura**

Japan

**Ryota Nakaoke**

Japan

**Kazuhiro Nakayama**

Japan

**Xiaopeng Ning**

United States of America

**Elizabeth Nisbet**

Canada

**Takayuki Nishimura**

Japan

**Hiroko Noto**

Japan

**Hisayoshi Ogata**

Japan

**Kumiko Ohara**

Japan

**Jun Ohashi**

Japan

**Hiroki Oota**

Japan

**Takuya Osawa**

Japan

**Jaeseok Park**

Korea, South

**Woojin Park**

Korea, South

**Giselle Passos**

Brazil

**Stephane Perrey**

France

**Alice Mado Proverbio**

Italy

**Sergey Rudnev**

Russian Federation

**Otto Sanchez**

United States of America

**Jerome Sarris**

Australia

**Reiko Sasaki**

Japan

**Yee**-**How Say**

Malaysia

**Yoshihiro Shimomura**

Japan

**Gwanseob Shin**

Korea, South

**Won Sop Shin**

Korea, South

**Shuichiro Shirakawa**

Japan

**Nami Someya**

Japan

**Su**-**Young Son**

Japan

**Yoshiaki Sone**

Japan

**Koji Sugiyama**

Japan

**Takayoshi Takahashi**

Japan

**Yuji Takasaki**

Japan

**Chiaki Tanaka**

Japan

**Nigel Taylor**

Australia

**Takuro Tobina**

Japan

**Fumiharu Togo**

Japan

**Kumpei Tokuyama**

Japan

**Yuko Tsunetsugu**

Japan

**Hitoshi Wakabayashi**

Japan

**De Yun Wang**

Singapore

**Shigeki Watanuki**

Japan

**Matthias Wieser**

Germany

**Jong Min Woo**

Korea, South

**Shuping Xiong**

Korea, South

**Tamaki Yamada**

Japan

**Kazuhiko Yamamoto**

Japan

**Sakae Yamamoto**

Japan

**Eric Yiou**

France

**Miyo Yokota**

United States of America

**Takeshi Yoneshiro**

Japan

**Susana Zeni**

Argentina

**Dingguo Zhang**

China

